# The Roles of the LIM Domain Proteins in *Drosophila* Cardiac and Hematopoietic Morphogenesis

**DOI:** 10.3389/fcvm.2021.616851

**Published:** 2021-02-11

**Authors:** Meihua She, Min Tang, Tingting Jiang, Qun Zeng

**Affiliations:** ^1^Department of Biochemistry and Molecular Biology, College of Hengyang Medical, University of South China, Hengyang, China; ^2^Affiliated Nanhua Hospital, University of South China, Hengyang, China

**Keywords:** *Drosophila melanogaster*, LIM domain, cardiac, hematopoietic, development

## Abstract

*Drosophila melanogaster* has been used as a model organism for study on development and pathophysiology of the heart. LIM domain proteins act as adaptors or scaffolds to promote the assembly of multimeric protein complexes. We found a total of 75 proteins encoded by 36 genes have LIM domain in *Drosophila melanogaster* by the tools of SMART, FLY-FISH, and FlyExpress, and around 41.7% proteins with LIM domain locate in lymph glands, muscles system, and circulatory system. Furthermore, we summarized functions of different LIM domain proteins in the development and physiology of fly heart and hematopoietic systems. It would be attractive to determine whether it exists a probable “LIM code” for the cycle of different cell fates in cardiac and hematopoietic tissues. Next, we aspired to propose a new research direction that the LIM domain proteins may play an important role in fly cardiac and hematopoietic morphogenesis.

## Introduction

The *Drosophila melanogaster* has emerged as a powerful model for studying heart development and cardiac diseases. Using the tools of both classical and molecular genetics, the study of the phylogeny of fly heart has been influential in the denomination of the primary signaling events of cardiac area formation, cardiomyocyte specification, and the formation of the functioning heart tube (1). Several studies that are underway may take advantage of the fruit fly as an *in vivo* model for exploring genes involved in cardiac formation. Numerous mutations in conserved hereditary pathways have been found, including those commanding development and physiology of the heart (2). The function and expression pattern of the homeobox transcription factor *Tinman* in *Drosophila* and its vertebrate homologous *NKX2.5* are remarkably similar in the embryology of heart tube morphogenesis and heart function, and this strikingly provides the compelling evidence that heart development is controlled by conserved homologous pathways in both invertebrates and vertebrates (2–6). After that, many transcription factors, and signal pathways involved in the cardiac specification has been elucidated. Pnr is essential in the mesoderm for initiation of cardiac-specific expression of tinman and for specification of the heart primordium (7). Wnt signaling activated by Wg, together with Dpp, is required for specification of cardiac progenitor cells in early heart development (8). These basic determinants of cardiogenesis in the fly play elementary roles in both the immature cardiac specification and heart function in the adult fly and human (4).

Heart progenitors of flies are bilaterally symmetrical, distributed in most of dorsal regions of the mesoderm in *Drosophila* (9). During cardiogenesis, these progenitors migrate to the dorsal midline and form the heart tube, a stretchable linear tube, which is consisted of two different types of cells, *ie*. contractile myocardial cells in the inner layer and non-contractile pericardial cells. Pericardial cells are the excretory cells, called pericardial nephrocytes (10, 11). *Drosophila melanogaster* also shares with vertebrates the basic regulatory mechanisms and genetic control of hematopoiesis, making it a good model for solving outstanding problems in blood development (12, 13). Lymph organ, the hematopoietic system in *Drosophila*, which produces the hemolymph cells, is derived from the cardiac progenitors, some genes that affect heart development may also be involved in regulating the differentiation of blood cells (14). *Drosophila* has three classes of blood cells: plasmatocytes, lamellocytes, and crystal cells. The development of crystal cells, a non-phagocytic cell, is similar to the vertebrate erythroid development in the early phase (15, 16). Despite its simple structure, the fly heart has recently emerged as an admirable model system for dissecting complicated pathways that determine the fate of cardiac cells and studying the physiological functions of the adult heart (17).

LIM is a small protein-protein synergism domain containing two zinc fingers (18). LIM domains are identified in a dissimilar group of proteins with a wide variety of biological roles, including gene expression regulation, cell fate determination, cytoskeleton organization, tumor formation, and embryonic development (19). LIM domains function as adaptors or scaffolds to support the assembly of multimeric protein complexes *via* interacting with alteration protein partners. LIM domains generally have about 50–60 amino acids and are characterized by two highly conserved zinc finger motifs. These zinc fingers contain eight conserved residues, generally with cysteines and histidines coordinately binding to two zinc atoms. The consensus sequence of LIM domain has been defined as C-X(2)-C-X(16, 23)-H-X(2)-[CH]-X(2)-C-X(2)-C-X(16,21)-C-X(2,3)-[CHD] (where X denotes any amino acid) (18–20).

In this review, Firstly, we found a total of 75 proteins encoded by 36 genes have LIM domain in *Drosophila melanogaster* by the tools of SMART, and most proteins with LIM domain locate in lymph glands, muscles system, and circulatory system according to FLY-FISH, and FlyExpress. Secondly, we summarized functions of four LIM domain proteins in the development and physiology of fly heart and hematopoietic systems. Lastly, we aspired to propose a new research direction that other LIM domain proteins may play an important role in fly cardiac and hematopoietic morphogenesis. Based on the expression parrerns, and biological roles of the LIM domain, we speculated that it has an important function involved in *Drosophila* heart development, heart function, and/or hematopoietic morphogenesis. Therefore, this review focus on the roles of LIM domain proteins in *Drosophila* cardiac and hematopoietic morphogenesis.

### Bioinformatics Analysis of *Drosophila* LIM Domain Proteins

**Figure 1:**
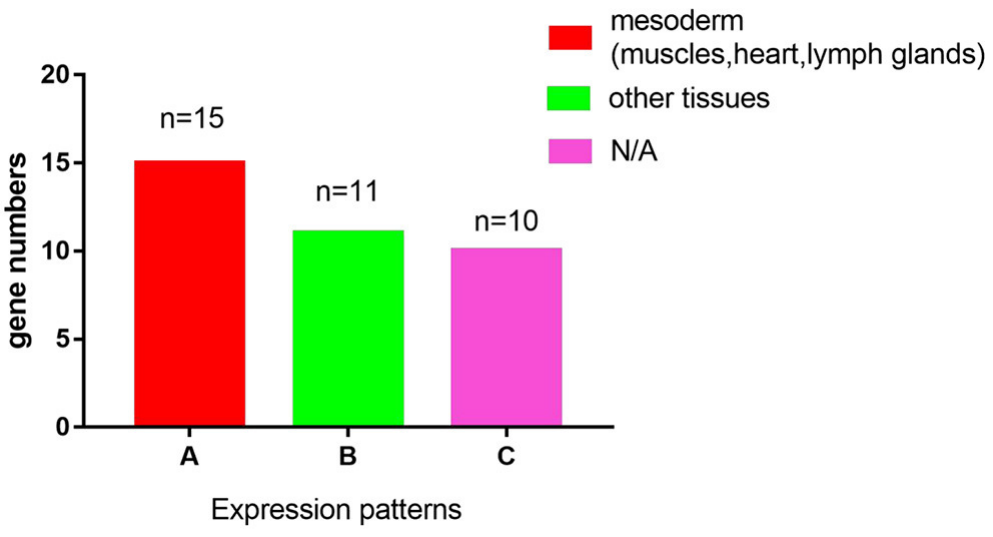
A graphic overview of the expression patterns of the 36 LIM genes in *Drosophila melanogaster*. According to the FLY-FISH or FlyExpress databases, there are 15 LIM genes expressed in mesoderm including muscles, heart, or lymph glands (Columm A). It indicates that 11 LIM genes are expressed in other non- Columm A tissues (Columm B). The expression patterns of the remaining 10 LIM genes are unclear (Columm C).

**Figure 2:**
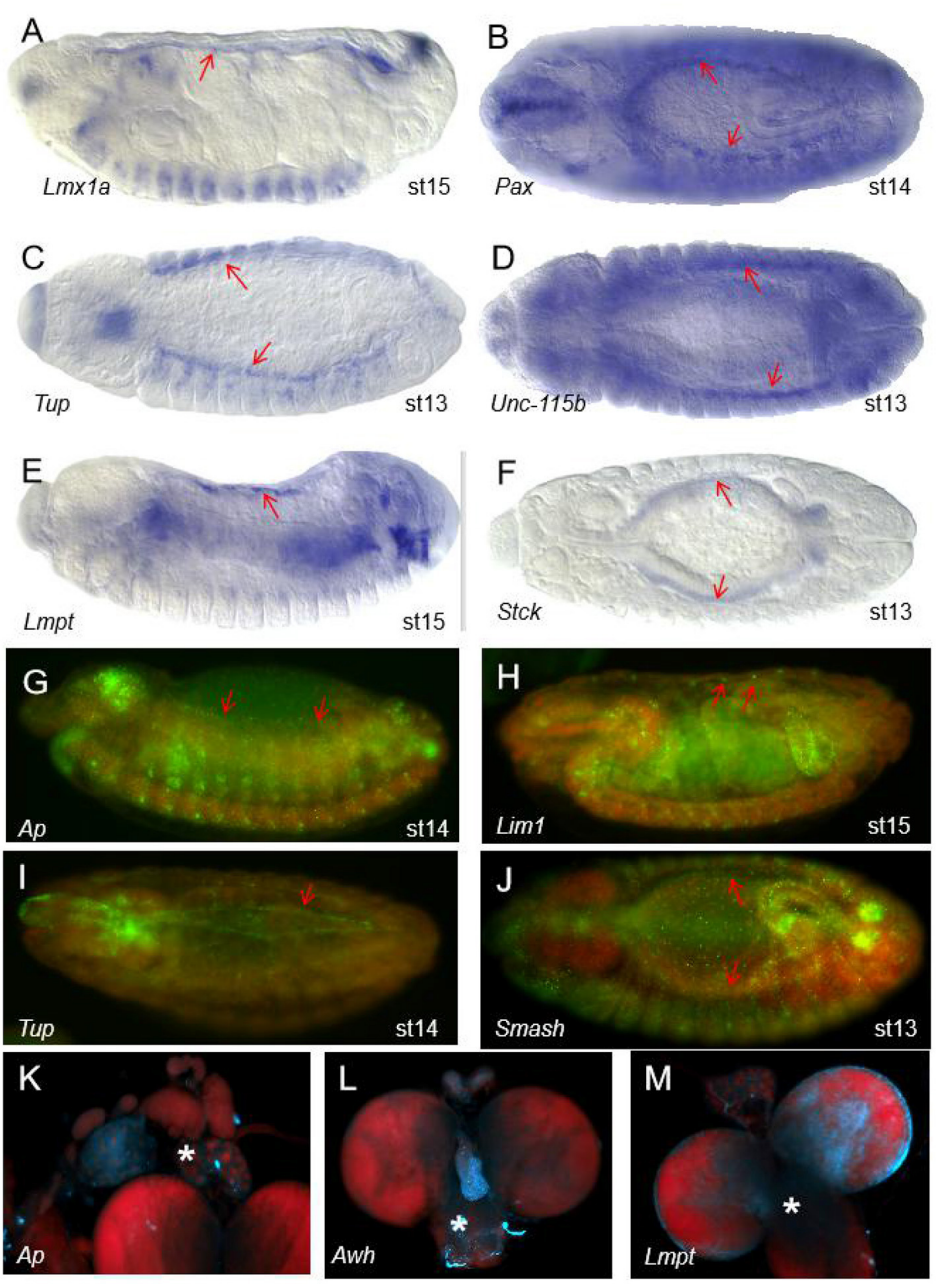
The expression patterns of some of the 36 LIM genes in *Drosophila melanogaster*. **(A–F)** Expression patterns of some genes with the LIM domain in wild type (WT) Drosophila embryos from the FlyExpress (ISH) database. The red arrows represent the heart region of the fruit fly embryo. Embryos are at stage 13–15 of development. Lmx1a **(A)**, Pax **(B)**, Tup **(C)**, Unc-115b **(D)**, Lmpt **(E)**, and Stck **(F)** are expressed in Drosophila heart tissue. **(G–J)** Expression patterns of some genes with the LIM domain in Drosophila embryos in the Fly-FISH database. The red arrows represent the heart region of the fruit fly embryo. Embryos are at stage 13–15 of development. Ap **(G)**, Lim1 **(H)**, Tup **(I)**, and Smash **(J)** are expressed in Drosophila heart tissue. Embryo colors: Red/DNA, Green/RNA. **(K–M)** The area of the lymph glands in the anatomy. Expression patterns of some genes with the LIM domain in the third larvae of Drosophila in the Fly-FISH database. The white asterisk represents the lymph gland region of the fruit fly larvae. Ap **(K)**, Awh **(L)**, and Lmpt **(M)** are expressed in Drosophila lymph gland. Larval colors: Red/DNA, Blue/RNA.

SMART (Simple Modular Architecture Research Tool) is a web resource for the identification and annotation of protein domains and the analysis of protein domain architectures (http://smart.embl.de) (21, 22). We used the SMART tool to identify proteins containing the LIM domain in *Drosophila melanogaster*. It is expected that a total of 36 genes encoding 75 proteins with the LIM domain in *Drosophila melanogaster* (Table 1). According to the characteristics of protein domains, LIM-domain proteins were classified into three types, LIM proteins, which include the LIM domain and other protein structures; LIM-only types with only the LIM domain; and LIM-homeodomain (LIM-HD) types, which include the LIM domain and homeodomain domain. These genes participate in the biological functions of various organs and tissues such as muscle, heart, lymph gland, nervous system, eye development, and leg morphogenesis, and so on (Table 1). Then we evaluated the expression regions of these genes in fruit flies by two databases. One is Fly-FISH (http://fly-fish.ccbr.utoronto.ca/), a database documents the expression and localization patterns of *Drosophila* mRNAs at the cellular and subcellular level during early embryogenesis and third instar larval tissues (**Figures 2G–M**) (23, 24). The other is FlyExpress (http://www.flyexpress.net/), a freely approachable online knowledgebase offering users an occasion to investigate and analyze expression patterns of developmental genes in *Drosophila* embryogenesis (**Figures 2A–F**) (25–27). According to the FLY-FISH or FlyExpress databases, there are 15 LIM genes expressed in mesoderm including muscles, heart, or lymph glands (Figure 1 Columm A). It indicates that 11 LIM genes are expressed in other non-Columm A tissues (Figure 1 Columm B). The expression patterns of the remaining 10 LIM genes are unclear (Figure 1 Columm C). From these two databases, we perceived that some of the 36 genes were expressed in the heart (Table 1 and Figures 2A–J), lymph gland (Table 1 and Figures 2K–M), muscle, CNS, and amnioserosa (Table 1), and so on. In general, around 41.7% *Drosophila* LIM domain genes were enriched expressed in mesoderm including muscles, heart, or lymph glands (Figure 1 Columm A), and this signified that these genes might have a important role in heart, and/or hematopoietic development. In the accompanying sections, we introduced some specific LIM domain proteins, tailup, mlp84B, radish, and Beadex, which have been confirmed by scientists, play roles in cardiac, and/or hematopoietic morphogenesis. However other LIM domain genes, which enriched expressed in heart, lymph glands, or other tissues, whether it is related to heart development, heart function, or hematopoietic morphogenesis remains to be further studied. In a word, we provide a new idea for the future research.

**Table 1:** List of the various transcripts and proteins with LIM domain in *Drosophila melanogaster*.

**FlyBase ID**	**gene**	**UniProt IDs**	**FLY-FISH**	**FlyExpress**	**LIM-type**
FBgn0263934	*Esn*	A0A0B4KED7 A0A0B4KEK5	N/A	N/A	LIM protein
FBgn0013751	*Awh*	M9PEI5	Brain, Ectoderm, Optic lobes, primordium, Posterior spiracles, Segmentedpattern, lymph glands	N/A	LIM-homeodomain (LIM-HD)
FBgn0259209	*Mlp60A*	B7YZP9	Muscle, Posterior spiracles	Muscle system, Dorsal prothoracic, Pharyngeal muscle,	LIM-Only
FBgn0263346	*Smash*	A0A0B4K615	CNS, Dorsal vessel, Ectoderm, Head, Hindgut, muscle, Visceral mesoderm, Segmented pattern	Dorsal apodeme, Dorsal epidermis, Hindgut	LIM protein
FBgn0053208	*Mical*	Q86BA1, A0A0B4K703, A0A0B4K6D5, A0A0B4K6N6, A0A126GUS6, A0A126GUS4	Blastoderm nuclei, Yolk nuclei, Zygotic	Midgut, Muscle system, Morsal prothoracic, Pharyngeal muscle	LIM protein
FBgn0036333	*Mical-like*	Q9VU34	API	Malpighiantubule, Midgut, Trunk mesoderm	LIM protein
FBgn0265991	*Zasp52*	A1ZA47, A0A0B4KER6, A0A0B4KEW3, A0A0B4KFR8, A0A0B4LGL0, C1C3E7 G3JX28, G3JX25 G3JX27, G3JX29 G3JX30, G3JX31, G3JX32 G3JX33	Amnioserosa, API, CNS, Midgut, Segmented pattern	Amnioserosa	LIM protein
FBgn0051352	*Unc-115a*	A0A0B4LGY0 E1JIH3	API, Ectoderm, Neuroblasts	N/A	LIM protein
FBgn0260463	*Unc-115b*	Q8INN7 A0A126GUR5	N/A	Amnioserosa, Mucsle, Mesoderm, Head	LIM protein
FBgn0003090	*Pk*	A1Z6W3, Q3YNB2	N/A	N/A	LIM protein
FBgn0034223	*Tes*	A1ZAT5	Zygotic	N/A	LIM protein
FBgn0041789	*Pax*	Q9BPQ9, Q966T5, Q2PDT4 Q9VIX2	N/A	Cardiac mesoderm, Muscle system	LIM-Only
FBgn0063485	*Lasp*	Q8I7C3 M9NDA6 M9NFH3	CNS, Ectoderm	CNS	LIM protein
FBgn0283712	*LIMK1*	Q8IR79, E1JJM7	Ubiquitous	N/A	LIM protein
FBgn0250819	*CG33521*	H9XVQ0	Muscle system,	Muscle system	LIM-Only
FBgn0261565	*Lmpt*	Q8IQQ3, Q8MYZ5, M9MRX0, Q9VVB5	Intestine, Muscles, PNS, Ring gland, Sensory system, Lymph Glands,	Muscle system, Tracheal system, Amnioserosa, Mesoderm	LIM protein
FBgn0026411	*Lim1*	Q9V472	Brain, CNS, Gut, Tracheal system,	Hypopharynx, Vent nerve cord,	LIM-HD
FBgn0002023	*Lim3*	Q9VJ02, M9PD53	CNS, Ventral nerve cord	Head	LIM-HD
FBgn0265598	*Beadex*	Q8IQX7, M9PI02, M9PHX4	Intestine, Brainlobes, Ovary, Testis	N/A	LIM-Only
FBgn0267978	*Ap*	P29673	Mesoderm, Brain, CNS, Tracheal system, Lymph Glands, Segmented pattern	N/A	LIM-HD
FBgn0050147	*Hil*	Q0E908	N/A	N/A	LIM protein
FBgn0014863	*Mlp84B*	Q24400	N/A	Muscle system	LIM-Only
FBgn0051624	*CG31624*	Q2XYF8	N/A	N/A	LIM-Only
FBgn0085354	*CG34325*	Q9VXC1	Ubiquitous	N/A	LIM-Only
FBgn0050178	*CG30178*	Q8MLQ2	Ubiquitous	N/A	LIM-Only
FBgn0020249	*Stck*	Q8IGP6	Ubiquitous	Visceral muscle	LIM protein
FBgn0011642	*Zyx*	Q8T0F5 Q9N675	Mesoderm	N/A	LIM-Only
FBgn0051988	*CG31988*	Q8T0V8	N/A	N/A	LIM-Only
FBgn0051624	*CG31624*	Q2XYF8	N/A	N/A	LIM-Only
FBgn0039055	*Rassf*	Q9VCQ7	N/A	N/A	LIM protein
FBgn0003896	*Tup*	Q9VJ37	Amnioserosa, API, Foregut, Head, Tracheal system, Muscles, Dorsal vessel	Amnioserosa, Leading edge cell, CNS, Dorsal epidermis, Embryonic brain, sophagus, Foregut, Stomatogastric	LIM-HD
FBgn0032196	*CG5708*	Q9VL21	N/A	NA	LIM-Only
FBgn0036274	*CG4328*	Q9VTW3	Ventral nerve, cord,CNS	NA	LIM-HD
FBgn0052105	*Lmx1a*	Q9VTW5	Ventral nerve cord,CNS	CNS, Trunk mesoderm, Longitudinal visceral mesoderm	LIM-HD
FBgn0030530	*Jub*	Q9VY77	N/A	N/A	LIM-Only
FBgn0265597	*Rad*	X2JBI1X2JDH0	N/A	N/A	LIM protein

*Proteins containing the LIM domain in Drosophila melanogaster were predicted by using the SMART. We found there are a total of 36 genes encoding 75 proteins with the LIM domain in Drosophila melanogaster. These genes play roles in a variety of biological functions, including muscle, heart, lymph gland, nervous system, eye development, and leg morphogenesis, and so on. According to the characteristics of protein domains, LIM proteins were classified into three types, LIM protein types, LIM-only types, LIM-homeodomain (LIM-HD) types*.

### LIM Homeodomain Transcription Factor *tailup* Is Required for Normal Heart and Hematopoietic Organ Formation in *Drosophila*

Fly LIM homeodomain transcription factor *tailup* (*Tup*), a homologous gene of *Islet1* in vertebrate, belongs to LIM-HD type, expresses in all cardiac cells (CC) and pericardial cells (PC) of the heart tube as well as lymph glands hematopoietic organs (28–30), which is not in line with the data in Table 1, we think it didn't be detected, or the probes did not work in the two databases (FLY-FISH, and FlyExpress). In vertebrates, the homologous gene *Islet1* is also a cardiac progenitor marker (31), and many of the heart formation processes are regulated by the transcription factor *Islet1* (32). It is reported that, in *Tup* mutant fly embryos, heart tubes showed misaligned cardio-blasts and lost most lymph glands and pericardial cells (33). This indicated *Tup* participated in fly heart development. While irregular morphology of the heart with hypertrophied lymph glands has been observed when *Tup* was overexpressed in fly mesoderm (33). This indicated *Tup* also participated in fly hematopoietic organ formation. *Tup* is an entrant in the regulatory network controlling dorsal vessel morphogenesis and hematopoietic organ formation. *Tup* can be considered to be a main upstream regulator of genetic and cellular events controlling lymph gland formation (33). For example, it is reported that Tup was an upstream regulator of *Hand* in these developmental processes. Tup can recognize and bind to two DNA response sequences in the enhancer of the *Hand*, which has been proven to be essential for the lymph gland cells, pericardial cells, and cardiac cells (34). Also, Tup is a regulator of *srp* and *odd*, which play an essential role in hematopoietic progenitor cell specification (33, 35). *Drosophila* LIM-HD domain protein Tup was specifically expressed in heart lymph gland tissue and had a unique role in heart development. LIM-domain factors affiliated to cooperate with the adaptor snippet Chip/Ldb1 to form higher-order protein complexes to determine gene expression (36). In a word, *Tup* interacted with Chip/Ldb1 to regulate some ingenious factors, such as *Even-skipped* (*Eve*), *Hand*, and *Odd*, involved in cardiac cell specification and differentiation (37–40). As we known, the GATA factors *pnr* and *srp* directly activate *Hand* in cardioblasts, pericardial nephrocytes and hematopoietic progenitors, *Hand* is initially expressed weak and segmental in cardiac cells, but soon becomes strong in most cardioblasts and pericardial cells from embryo in stage13 to adult heart. The pattern expression of *hand* is controlled by a 513bp enhancer between exons 3 and 4 of the *Hand* gene, which contained DNA sequence with Tinman- and GATA-binding sites (41).

Due to the expression patterns and functions of *Tup* in fly heart, as well as some other LIM-HD type genes listed in the Table 1, which expressed in the mesoderm at early stage, it is implied that LIM-HD domain proteins might regulate the differentiation and specialization of cardiac progenitor cells in early cardiac development, and interacte with Chip/Ldb1 to determine normal heart and hematopoietic organ formation in *Drosophila* (36). Also, some genes involved in heart development could be the targets of LIM-HD domain proteins.

### *Mlp84B*, a Muscle LIM Protein, Is Essential for Cardiac Function in Drosophila

*Mlp84B*, a muscle LIM protein (MLP), belongs to the LIM-only type, locates at the Z-disc of sarcomeres (42, 43). The human MLP gene has been represented to be a key player in the stretch-sensing response, and its mutant is associated with hypertrophic and dilated cardiomyopathy (44). In mice, the LIM domain protein CRP2, a *Drosophila* Mlp84B homolog, expressed in the vascular system, especially in smooth muscle cells. CRP2 knock-out mice exhibit some mild hypertrophy phenotype in their cardiac ultrastructure (45). *Mlp84B*, co-localizes with α*-actinin* in the heart from late embryonic stages to adulthood in *Drosophila*, and is essential for *Drosophila* lifespan and cardiac function (46, 47). The shortened lifespan was found in *Mlp84B* knockout flies, and with a dramatically decreased lifespan in knockdown flies by the cardiac driver tinCΔ4-Gal4, which are due to the role of Mlp84B in cardiac function. *Mlp84B* mutant flies appeared bradycardia and heart rhythm abnormalities without obvious organic phenotype (46). We believe that MLP proteins may play a critical role in the differentiation of cardiac muscle and contribute to proper cardiac function by directing cardiac muscle structure development. It is known that *dMEF2*, an invertebrate member of the Myocyte Enhancer Factors family of vertebrate myocyte, has MADS-box and MEF2 domains which assisted its dimerization and DNA binding, plays an essential role in muscle differentiation (48). Due to the six dMEF2 binding sites found in the *Mlp84B* locus, *dMEF2* is necessary for the expression of *Mlp84B* in all the muscle tissues including skeletal, cardiac, and smooth muscles (48). Collectively, it suggested that *Mlps* may be targets of dMEF2 proteins that contribute to cardiomyocyte differentiation and function. We hypothesized that LIM domain genes expressed in muscle tissue are likely regulated by MEF2, which determines the differentiation of the three types of muscle cells. We predicted LIM genes expressed in heart muscle tissue, such as *Mlp60A, Smash, Lmpt*, etc (see Table 1), might be essential for the differentiation of cardiomyocyte.

### The *Drosophila radish* Gene Is Required for Cardiac Pacemaker

**Figure 3:**
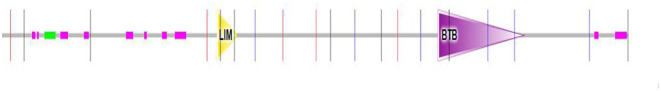
Domains within *Drosophila melanogaster* protein Radish. Yellow triangle box represents the LIM domain region, the LIM domain of Radish was located at positions 568 to 622 amino acids.

The *Drosophila radish (Rad)* gene is required for cardiac function (49). It is known that *Rad*, encoding a protein with a POZ domain and a Rap GTPase activating protein domain, involved in regulating anesthesia-resistant memory and heart contraction function (49, 50). Interestingly, we found that Rad is also a member of the LIM protein family, based on the predictions of SMART tool (Table 1). We also analyzed the domains within *Drosophila melanogaster* protein Radish, the LIM domain was located at positions 568 to 622 amino acids (Figure 3). Conveniently, there is no expression data presented for Rad in Table 1, further studies are needed to confirm whether it is expressed in heart tissue. The exceptional *Rad* mutants, which effected memory and retention, substantially lessened heart rate and rhythms. The heart rate of *Rad* mutants was significantly lower than that of wild mutants at different temperatures (49). Besides, these mutants were insensitive to both serotonin and norepinephrine. In a word, *Rad* mutations were closely associated with bradycardia in fly. Its effect on heart function is likely mediated via neurotransmitter release (49). While the molecular mechanism by which the *Rad* regulates heart function remained ambiguous, *Rad* likely effects the heart modulatory pathway (49). We knew that muscle LIM protein Mlp84B, mainly located at the Z-line boundary, maintained the structural integrity of muscles by interacting with α-actinin, and D-titin was essential for cardiac function (47, 51). We hypothesized the LIM proteins, such as Rad, if it is expressed in the heart muscle tissue, it maybe also interacted with the modular protein present in muscles to regulate the structure of the heart muscles and thus affected the function of the heart. So LIM proteins not only regulate cardiac development but also affect cardiac function.

### *Beadex*, a *Drosophila* Homolog of LIM Domain Only 2 (*LMO2*), Functions in Hematopoiesis

The lymph organ, which produces the hemolymph cells, is derived from the cardiac progenitors, some of the genes that affect heart development also involved in hematopoiesis. Tup, a regulator of *srp* and *odd* expression in lymph gland cells and pericardial cells (33, 35), is a typical example. So we think that in addition to affecting heart function and heart development, LIM proteins also regulate blood cell formation in *Drosophila*. There are three classes of blood cells *in Drosophila*: plasmatocytes, lamellocytes, and crystal cells (17, 52). The development of crystal cell, a non-phagocytic cell, is similar to that of vertebrate erythroid in the early phase (53). In Table 1, we found that *Beadex*, a *Drosophila* homolog of LIM domain only 2 (*LMO2*), is expressed in hemocytes, and was reported to effect the numbers of crystal cells after their specification (54). Morever, in vertebrate, *LMO2* is necessary for the emergence of hematopoietic stem cells during ontogeny and angiogenesis by modeling a complex with the LIM domain-binding protein 1, and binding to the E-box and GATA transcription factors (55, 56). In fly, overexpression of *Beadex* in crystal cell lineage leading to a dramatic increase in the number of crystal cells. Meanwhile, a knockdown of *Beadex* resulted in a significant decrease in the number of crystal cells (3). This indicated *Beadex* is involved in the proliferation of blood cells in fly. *Drosophila* GATA factor *pannier* (*Pnr*), a known interacting colleague of *Beadex*. Gain of function (GOF) of both *pnr* and *Beadex* in fly, the crystal cell counts were consistent with that of *pnr* GOF alone, and GOF of *Beadex* and loss of function (LOF) of pnr in fly, the crystal cell counts were similar to that of *pnr* LOF alone, therefore, the misexpression of *pnr* masked the effect of *Beadex* on the number of crystal cells. So, *pnr* has genetic interactions with *Beadex* during the development of crystal cell (3, 57, 58). *Pnr*, as a downstream molecular, can bind to cofactor U-shaped (Ush) to inhibite crystal cell development (3, 59). Thus, *pnr* is also supposed to interfere in the development of crystal cells. That is to denote, LIM proteins might contribute to hematopoietic development through the GATA transcription factor. According to the Fly-FISH database, we also found that some LIM genes expressed in the lymph glands of *Drosophila* (Table 1 and Figures 2K–M). Combined with the study on the role of *Beadex* in the hematopoietic system in *Drosophila*, we speculate that LIM proteins may also have a role in the hematopoietic system of *Drosophila*.

## Perspectives

This review summarized the role and molecular mechanisms of LIM transcription factors of *Drosophila* during cardiovascular development. The *Drosophila melanogaster* has emerged as a powerful model for studying heart development and cardiac diseases, as well as an excellent model for human cardiac development and diseases (17, 52). LIM domains are identified in diverse groups of proteins with a wide variety of biological functions, including gene expression regulation, cell fate determination, cytoskeleton organization, tumor formation, and heart development (18–20). Just as LIM related factors arise to be conserved in the arrangement of cardiac development and physiology in the animal kingdom from fly to human.

Through analysis of the bioinformatics database of *Drosophila* LIM domain transcription factors, a total of 36 genes encoding 75 proteins with the LIM domain in *Drosophila melanogaster* were found. We divided LIM proteins into three categories, LIM protein type, LIM-only type, and LIM-HD type, according to the characteristics of protein domains. LIM domain proteins can be either transcription factors or structural proteins to regulate cardiac and hematopoietic morphogenesis. As a transcription factor, such as *tup*, can recognize and bind to two DNA response sequences in the enhancer of the *Hand*, and act as a regulator of *srp* and *odd* to determines the differentiation of lymph gland cells, cardiac cells, and pericardial cells. As a structural protein, such as Mlp84B, is the cytoskeletal protein, directly affects muscle structure and function. From Fly-FISH and FlyExpress (23–27), we knew that some of LIM domain mRNAs were located in lymph glands, muscles system, and circulatory system, which inferred that LIM domain genes might have roles in cardiac and hematopoietic morphogenesis. It would be attractive to dissect whether it exists a probable LIM code for the cycle of different cell fates in cardiac and hematopoietic tissues, and this hypothesis needs to be better supported in future studies. In this review, we aspired to proposed a research direction that the LIM domain genes may play important roles in cardiac development and hematopoiesis. Cardiomyocytes are a type of muscle cells, and dMef2 regulates the differentiation of cardiomyocytes, therefore, we hypothesized that one possible mechanism is that LIM domain genes can act as the targets of dMef2 in heart tissue, regulate the differentiation of cardiomyocytes, interact with Chip/Ldb1 to determine normal heart and hematopoietic organ formation in *Drosophila*, and participate in hematopoietic development through GATA transcription factor.

## Author Contributions

MS and QZ wrote and edited the manuscript together. MT and TJ generated the figures and table together. All authors contributed to the article and approved the submitted version.

## Conflict of Interest

The authors declare that the research was conducted in the absence of any commercial or financial relationships that could be construed as a potential conflict of interest.
